# A chronology of ratios between black smoke and PM_10_ and PM_2.5_ in the context of comparison of air pollution epidemiology concentration-response functions

**DOI:** 10.1186/s12940-017-0252-2

**Published:** 2017-05-03

**Authors:** Mathew R. Heal, Iain J. Beverland

**Affiliations:** 1 School of Chemistry, University of Edinburgh, David Brewster Road, Edinburgh, EH9 3FJ UK; 2 Department of Civil and Environmental Engineering, University of Strathclyde, James Weir Building, 75 Montrose Street, Glasgow, G1 1XJ UK

**Keywords:** Black smoke, PM_10_, PM_2.5_, Epidemiology, Air pollution, Exposure

## Abstract

**Background:**

For many air pollution epidemiological studies in Europe, ‘black smoke’ (BS) was the only measurement available to quantify ambient particulate matter (PM), particularly for exposures prior to the mid-1990s when quantification via the PM_10_ and/or PM_2.5_ metrics was introduced. The aim of this work was to review historic BS and PM measurements to allow comparison of health concentration-response functions (CRF) derived using BS as the measure of exposure with CRFs derived using PM_10_ or PM_2.5_.

**Methods:**

The literature was searched for quantitative information on measured ratios of BS:PM_10_, BS:PM_2.5_, and chemical composition of PM; with specific focus on the United Kingdom (UK) between 1970 and the early 2000s when BS measurements were discontinued.

**Results:**

The average BS:PM_10_ ratio in urban background air was just below unity at the start of the 1970s, decreased rapidly to ≈ 0.7 in the mid-1970s and to ≈ 0.5 at the end of the 1970s, with continued smaller declines in the 1980s, and was within the range 0.2–0.4 by the end of the 1990s. The limited data for the BS:PM_2.5_ ratio suggest it equalled or exceeded unity at the start of the 1970s, declined to ≈ 0.7 by the end of the 1970s, with slower decline thereafter to a range 0.4–0.65 by the end of the 1990s. For an epidemiological study that presents a *CRF*_BS_ value, the corresponding *CRF*_PM10_ value can be estimated as *R*_BS:PM10_ × *CRF*_BS_ where *R*_BS:PM10_ is the BS:PM_10_ concentration ratio, if the toxicity of PM_10_ is assumed due only to the component quantified by a BS measurement. In the general case of some (but unknown) contribution of toxicity from non-BS components of PM_10_ then *CRF*_PM10_ > *R*_BS:PM10_ × *CRF*_BS_, with *CRF*_PM10_ exceeding *CR*F_BS_ if the toxicity of the other components in PM_10_ is greater than the toxicity of the component to which the BS metric is sensitive. Similar analyses were applied to relationships between *CRF*_PM2.5_ and *CRF*_BS_.

**Conclusions:**

Application of this analysis to example published *CRF*_BS_ values for short and long-term health effects of PM suggest health effects from other components in the PM mixture in addition to the fine black particles characterised by BS.

**Electronic supplementary material:**

The online version of this article (doi:10.1186/s12940-017-0252-2) contains supplementary material, which is available to authorized users.

## Background

Prior to the 1990s concentrations of ambient airborne particulate matter (PM) in Europe were largely quantified by the darkness of the particulate material collected on filter papers. In this ‘Black Smoke’ (BS) method, the proportion of white light reflected from the sample (essentially the complement of the darkness) was converted to a mass concentration of PM using a standard equation [1, 2]. From the mid-1990s, ambient PM began to be quantified as PM_10_ and/or PM_2.5_, the total mass concentration of all particles within rigorously-defined size fractions, and these are now the usual measure of PM exposure for epidemiological studies [3]. However, for many studies investigating associations between historical air pollution and adverse health, BS values were the only exposure data available. For example, the United Kingdom (UK) Committee on the Medical Effects of Air Pollutants (COMEAP) [4] reported a meta-analysis of 29 time series showing a mean relative risk for cardiovascular premature mortality of 0.6% (95% CI: 0.4–0.7%) per 10 μg m^−3^ increment in short-term exposure to BS, whilst Janssen et al. [5] derived a pooled risk (from 7 single-city studies) of 0.90% (0.40–1.41%) for cardiovascular mortality, and 0.68% (0.31–1.06%) for all-cause mortality, per 10 μg m^−3^ increase in BS. There are fewer epidemiological studies of the long-term health effects of particulate air pollution but BS again features as a key metric of exposure [6–10]. The associations between concentrations of black particles and ill-health have been emphasised by the World Health Organisation [11] and others [12].

The concentration value assigned to a BS measurement is defined (for British BS) in British Standard 1969:1747:2 [1] and was established by weighing filter samples collected in parallel to the reflectance measurements. The Organisation for Economic Co-operation and Development (OECD) definition of BS is related by a simple multiplier: BS_OECD_ = BS_BRITISH_/0.85 [13]. The BS calibration curve was established from measurements in 1963 when smoke from residential, commercial and industrial coal burning was the dominant source of ambient PM in urban areas. At that time the concentrations of smoke-derived particles were so large that non-black particles (secondary inorganic material, sea salt, (re)suspended dust and soil, etc.) constituted a small proportion of the sampled PM. Since then, the nature of the ambient particle mixture has changed substantially and it has long been known that the BS ‘concentration’ obtained from the calibration curve does not equate to the total mass concentration of particles sampled (e.g. [14–16]). This raises the question of how to compare health coefficients expressed in terms of BS ‘concentrations’ relative to those expressed in terms of PM_10_ and PM_2.5_ concentrations.

If BS and PM_x_ (used here to mean PM_10_ or PM_2.5_) characterised completely different exposures to particulate air pollution then nothing could be inferred about the health response coefficient for PM_x_ from that for BS. However, BS and PM_x_ are both measures of a given inhaled particle mixture. BS values have been shown to correlate strongly with the elemental carbon (EC) component of PM (as derived by thermal methods) (e.g. [17–19]). BS is also quantitatively related to black carbon (BC), an alternative optically-derived measure of EC [2]. BS is therefore a good measure of the combustion-derived component of PM_x_. Some studies have also demonstrated correlation between BS (or filter darkness) and PM_x_ both temporally (e.g. [16, 20, 21]) and spatially [18, 22, 23] although ratios can also vary from location to location [24]. Given these physical relationships between BS and PM_x_, there must also be some relationship between BS and PM_x_ health response coefficients, albeit with this relationship dependent on assumptions about what components (size and chemical) of the inhaled PM mixture contribute to its toxicity.

In this work the published literature and datasets were searched to determine changes in BS:PM_x_ ratios from around 1970 to the early 2000s, when the majority of BS measurements in the UK were discontinued. The focus is on the BS metric (i.e. not filter absorbance, BC or EC measurements), and on urban background environments relevant to the populations underpinning epidemiological studies. Given the paucity of contemporaneous measurements, only broad trends in ratios can be derived. Nevertheless inferences can be made from these ratios about the relationship between a health response coefficient expressed as a function of BS concentrations with equivalent coefficients expressed as a function of PM_10_ or PM_2.5_ concentrations and consequently about the extent to which an adverse health outcome associated with inhaled PM is associated with the particle components characterised by BS.

## Methods and results

### Historic BS:PM_x_ ratios derived from gravimetric measurements

In the British BS method ambient air was sampled via an inverted funnel and short-length of copper tube through a separate 25-mm diameter Whatman No. 1 filter paper per 24 h period. The reflectance of the collected particle sample was subsequently measured using a white-light reflectometer and the percentage reflection converted to a daily-average BS mass concentration using a quartic calibration equation [1]. The *D*_50_ particle diameter cut-off for a standard BS sampler has been measured as 4.4 μm [25]. Thus, in 1963, when reflectance was originally calibrated against gravimetric measurements, BS concentrations equalled PM_4.4_ by definition. Consequently, BS:PM_2.5_ and BS:PM_10_ ratios at that time would have been >1 and <1, respectively. The BS:PM_10_ ratio would be around 0.8–0.9 if a value of 0.8–0.9 for the PM_4.4_:PM_10_ ratio is assumed. Evidence for this latter ratio, albeit relating to a later time period, is the average PM_4.4_:PM_10_ ratio of 0.85 reported by the UK Airborne Particles Expert Group [26] for unpublished data from Leeds in 1995, and the average PM_4_:PM_10_ ratio of 0.81 reported by Roosli et al. [27] for measurements in Switzerland in 1997 using Digitel DHA 80 high volume samplers.

Between May 1975 and April 1976, Ball and Hume [14] made weekday measurements of BS and TSP (total suspended particles) on the roof of the County Hall building in central London. For their annual dataset, the BS:TSP ratio was ≈ 0.5 (0.4 for summer, 0.6 for winter). From a detailed analysis of co-located TSP and PM_10_ measurements from the early 1980s, van den Muelen et al. [28] reported that average PM_10_:TSP ratios for western Europe were ≈ 0.7. If the PM_10_:TSP ratio in the mid-1970s was close to unity, then the Ball and Hume [14] data indicate a BS:PM_10_ ratio of ≈ 0.7.

The BS calibration curve was re-evaluated in 1979 and the PM mass concentration shown to be approximately double the value derived from application of the original calibration equation [15], i.e. in 1979, BS:PM_4.4_ ≈ 0.5 on average across the 5 sites and seasons investigated. The divergence in the quantitative relationship in this study (and that of Ball and Hume [14]) from the original calibration measurements in 1963 illustrates the rapid decline throughout this period of the contribution of black particles to PM due to implementation of smoke control measures. Using as above a PM_4.4_:PM_10_ ratio of 0.8–0.9, the Bailey and Clayton [15] measurements yield a BS:PM_10_ ratio of ≈ 0.4–0.45. Bailey and Clayton [15] also reported data from a sampling system with a single-stage impactor that indicated an average BS:PM_2_ ratio of 0.76. This latter cut point is close to the PM_2.5_ size fraction, indicating that at the time of these measurements BS:PM_2.5_ ≈ 0.7.

For measurements made in central Leeds for 3–4 months in 1982, Clarke et al. [29] reported data for BS, and from Sierra Model 245 automatic dichotomous samplers, showing average BS:PM_2.5_ and BS:PM_15_ ratios of 0.54 and 0.34 respectively. The latter indicates a BS:PM_10_ ratio of ≈ 0.3 if a PM_10_:PM_15_ ratio of ≈ 0.9 is assumed.

From a year of daily measurements in Bristol in 1993, average BS:PM_10_ ratio was 0.23 [30]. In a study of wintertime PM_10_ and black smoke concentrations across Europe in 1993/4 the median BS:PM_10_ ratio in Amsterdam was 0.35 (no data were collected in the UK in this study, hence Amsterdam was the city most representative for comparison with UK observations) [16]. The UK Quality of Urban Air Review Group reported mean daily ratios of BS:PM_10_ = 0.29 and BS:PM_2.5_ = 0.48 from co-located measurements at Birmingham Hodge Hill between Jan–Jun 1995 [31].

Daily BS, PM_10_ and PM_2.5_ (the latter two metrics quantified by Partisol gravimetric sampler) measured at the same urban background site in Edinburgh during 1999 and 2000 [32] had median (and interquartile range) daily ratios of 0.42 (0.27–0.60) for BS:PM_10_ and 0.80 (0.51–1.09) for BS:PM_2.5_. The higher BS:PM_2.5_ ratio in Edinburgh compared with the above data for Birmingham, Bristol and Leeds is likely a consequence of the relatively low PM_2.5_ to PM_10_ ratio at this Edinburgh site [32].

**Fig. 1:**
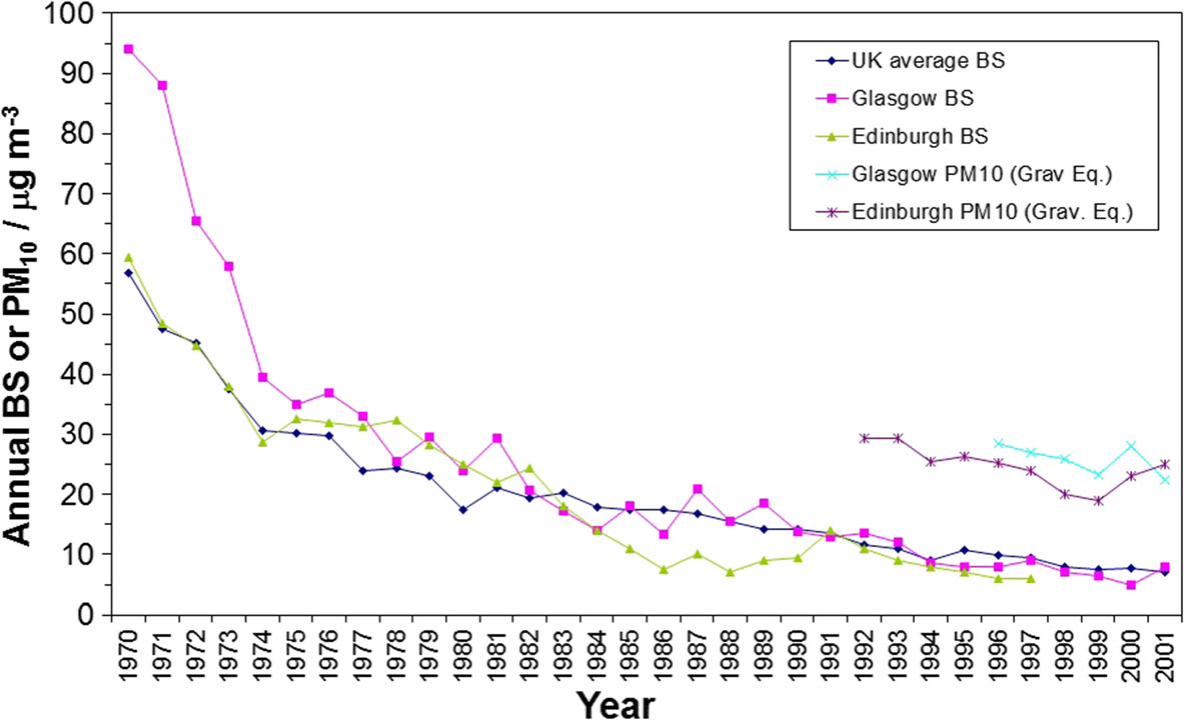
Annual average BS in Glasgow, Edinburgh and annual average for multiple sites across the UK for 1970–2001. The Glasgow and Edinburgh BS time series are averages across representative background sites in each city (sites EDI 10, 12 & 14 and GLA 51, 68, 95 & 98) operated at different times during the period covered. The UK-average BS time series was computed from sites operational in that year. Also shown are the annual average urban centre ‘gravimetric equivalent’ TEOM PM_10_ data for Glasgow and Edinburgh from the initiation of these measurements in the 1990s. All data obtained from the UK air quality data archive (http://uk-air.defra.gov.uk/data)

The ratio of BS to PM_10_ was examined in more detail for Glasgow and Edinburgh using data from the UK air quality data archive (http://uk-air.defra.gov.uk/data) for the period during the late 1990s and early 2000s when both were being measured (Fig. 1) (although in neither city were the measurements co-located). The BS time series were calculated by averaging data across 4 and 3 BS monitoring sites in Glasgow and Edinburgh respectively. The mean BS:PM_10_ ratios for the overlap periods in the mid to late 1990s in Fig. 1 were 0.30 and 0.29 for Glasgow and Edinburgh respectively using ‘gravimetric equivalent’ PM_10_ data. The equivalent BS:PM_10_ ratios were 0.38 and 0.38 using unadjusted Tapered Element Oscillating Microbalance (TEOM) PM_10_ data. BS trends in Glasgow and Edinburgh were broadly similar to trends in UK-average BS estimated from UK ‘smoke and SO_2_ network’ annual reports (http:/uk-air.defra.gov.uk) (Fig. 1). Co-located PM_2.5_ and PM_10_ measurements in the late 1990s from southern UK [33] and across Europe [34] indicate PM_2.5_:PM_10_ ratios generally in the range 0.6–0.8. Combining these ratios with BS:PM_10_ ratios of 0.3–0.4 suggests that BS:PM_2.5_ ratios at this time may have been in the range 0.4–0.65.

**Fig. 2:**
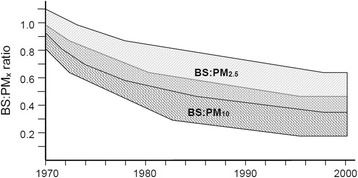
Schematic of estimated ranges (*shaded areas*) for BS:PM_10_ and BS:PM_2.5_ ratios in UK urban areas between 1970 and 2000 based on data from the observational studies discussed in the text. The *shaded* areas do not represent formal statistical confidence intervals. If the average size of the ‘*black*’ (i.e. *optically absorbing*) particles in the particle mixture was decreasing through this time period then the reduction in the proportion of *black* particles in the PM mixture may be slightly greater than the reduction in the numerical ratios between BS and PM_x_ illustrated in this figure (*see text for more detail*). The *middle shaded* area represents the overlap between the 2 sets of estimates

The broad trends in BS:PM_x_ ratios inferred from the literature described above are illustrated schematically in Fig. 2. In summary, the available data indicate the following general trend in average BS:PM_10_ ratio in urban background air: close to unity at the start of the 1970s; falling rapidly to ≈ 0.7 in the mid-1970s; ≈0.5 at the end of the 1970s; further smaller decline in the 1980s; within the range 0.2–0.4 in the 1990s. Direct data for estimation of BS:PM_2.5_ ratios were sparser, and indicate the following general trend: equal or exceeding unity at the start of the 1970s; a decline to ≈ 0.7 by the end of the 1970s; further decline through the 1980s; within the range 0.4–0.65 by the end of the 1990s.

### Historic BS:PM_x_ ratios derived from compositional measurements

BS:PM_x_ ratios can also be derived through estimation of historic PM composition, assuming that the majority of PM mass is due to the following components: ammonium sulphate, ammonium nitrate, sodium nitrate, sodium chloride, primary ‘dusts’, elemental carbon, organic carbon and water of hydration [35, 36]. However, historic measurements of PM chemical speciation back to the 1970s are very limited. Data are by far most abundant for sulphate. A summary of the estimates for the historical concentrations of the major chemical components within UK urban PM_2.5_ and PM_10_ is given in Table 1. A detailed description of the origin of the values assigned to these chemical components is provided in the Additional file 1.

**Table 1:** Summary of estimates of historical UK average urban BS concentrations, and of individual and summed component concentrations in PM_2.5_ and PM_10_ (μg m^−3^). Summations of components assume individual ranges correspond to 4 sd of uncertainty (i.e. that ranges approximate to a 95% confidence interval) and use standard formulae for combinations of uncertainties. The same approach is used to combine uncertainties in PM_2.5_:PM_10_ splits with the ranges of individual components. Detailed explanation of how these values are derived is given in the Additional file 1. The bottom two rows provide the BS:PM_x_ ratios implied by this speciated approach and as inferred from the direct measurements discussed in Section 2

PM component	1970s	1980s	Late 1990s	PM_2.5_/PM_10_ split	1970s	1980s	Late 1990s
	BS				BS		
	35	18	8		35	18	8
	PM_10_				PM_2.5_		
(NH_4_)_2_SO_4_	7–16	7–16	4–7	0.8–0.9	6–14	6–14	3–6
NH_4_NO_3_ + NaNO_3_	3–7	3–10	3–10	0.5–0.7	2–4	2–6	2–6
NaCl	2–5	2–5	2–5	0.2–0.4	0.5–1.5	0.5–1.5	0.5–1.5
Dust	2–4	2–4	2–4	0.2–0.4	0.5–1.5	0.5–1.5	0.5–1.5
EC	8–10	4–5	2–3	0.7–0.9	6–9	3–4	1–2
OM	13–37	6–19	3–9	0.7–0.9	10–30	5–15	2–7
Total PM_x_	44–70	33–50	21–31		31–53	23–36	13–20
BS:PM_x_ ratio (via speciated data)	0.5–0.8	0.35–0.5	0.25–0.4		0.65–1.1	0.5–0.8	0.4–0.6
BS:PM_x_ ratio (direct measurements)	≈0.7	≈0.5	≈0.3		0.7–1.1	0.5–0.8	0.4–0.65

The compositional estimates in Table 1 are expressed in Table 2 as the proportions of PM_10_ and PM_2.5_ in the 1970s and late 1990s that comprised: carbonaceous material; secondary inorganic material; and primary sea-salt and (re)suspended dust combined. The proportions estimated for PM_10_ in the late 1990s are consistent with the average proportions of approximately one-third each carbonaceous, secondary inorganic, and sea-salt plus dust reported for urban background PM_10_ in Birmingham and Glasgow in 2000 [37]. The concentrations of sea-salt, primary dust and secondary inorganic aerosol have remained about the same between the 1970s and late 1990s (a decrease in secondary sulphate in the latter was offset by an increase in secondary nitrate), but concentrations of carbonaceous material have decreased substantially. The proportions in Table 2 for PM_2.5_ in the late 1990s are likewise consistent with the average proportions of 0.4–0.5 primary carbonaceous, 0.4–0.5 secondary inorganic and 0.1–0.2 sea-salt/primary dust reported by the UK Air Quality Expert Group [37, 38] for urban background PM_2.5_ for the year 2000.

**Table 2:** Estimates of the proportions of carbonaceous, secondary inorganic, and sea-salt and dust material within UK urban PM_10_ and PM_2.5_, in the 1970s and late 1990s, derived from the individual component concentrations given in Table 1

chemical component	PM_10_		PM_2.5_	
	1970s	late 1990s	1970s	late 1990s
carbonaceous	0.5–0.7	0.3–0.4	0.6–0.8	0.3–0.5
secondary inorganic	0.2–0.4	0.3–0.4	0.25–0.4	0.4–0.6
sea-salt and dusts	0.1–0.15	0.2–0.3	0.05	0.05–0.15

Table 1 also presents the totals of the historical compositional concentrations, and compares BS:PM_x_ ratios estimated by this speciated approach to ratios estimated from the direct measurements of BS and PM_x_ reported in Section 2 and Fig. 1. The ranges of these estimates were quite large, but there was consistency between magnitudes of the BS:PM_x_ ratios derived in the two approaches.

## Discussion

BS and PM_x_ are both measures of inhaled particles, the latter the mass concentration of all particles in a specified size fraction, the former a measure of a sub-component of the PM_4.4_ size fraction but assigned a concentration value that does not equal the true concentration of that sub-component. The estimation of a concentration response coefficient for one metric from the concentration response coefficient derived for the other depends on the assumptions of where in the inhaled PM the toxicity lies.

Consider first the relationship between the concentration-response functions for BS (*CRF*_BS_) and PM_10_ (*CRF*_PM10_). The PM_4.4_ size fraction of the BS measurement is a subset of the PM_10_ size fraction. If uniform toxicity in all particle components within PM_10_ is assumed then the health risk expressed per unit increase in PM_10_ is the same as the health risk expressed per same unit increase in BS, i.e. *CRF*_PM10_ = *CRF*_BS_. If the toxicity of PM_10_ is assumed to result only from the component to which the BS metric is sensitive then *CRF*_PM10_ = *R*_BS:PM10_ × *CRF*_BS_ where *R*_BS:PM10_ is the BS:PM_10_ concentration ratio. In the general case of some (but unknown) contribution of toxicity from non-BS components of PM_10_ then *CRF*_PM10_ > *R*_BS:PM10_ × *CR*F_BS_, with *CRF*_PM10_ exceeding *CRF*_BS_ if the toxicity of the other components in PM_10_ is greater than the toxicity of the component to which the BS metric is sensitive.

Meaningful relationships can only be derived between *CRF*_PM2.5_ and *CRF*_BS_ if it is assumed that all the particles quantified by BS are within the PM_2.5_ size fraction, in which case the same analysis applies: if the toxicity of PM_2.5_ is assumed due only to the component to which the BS metric is sensitive then *CRF*_PM2.5_ = *R*_BS:PM2.5_ × *CRF*_BS_ where *R*_BS:PM2.5_ is the BS:PM_2.5_ concentration ratio; and in the general case of assumed (but unknown) toxicity in the other components of PM_2.5_ then *CRF*_PM2.5_ > *R*_BS:PM2.5_ × *CRF*_BS_, with *CRF*_PM2.5_ equalling *CR*F_BS_ if the toxicity of the other components in PM_2.5_ is the same as the toxicity of the component quantified by BS. If a proportion of particles of the BC component characterised by BS are in a size fraction greater than PM_2.5_ then it is not possible to link *CRF*_PM2.5_ and *CRF*_BS_ without knowledge of this proportion. However, it is likely that the major proportion of particles characterised by BS are within PM_2.5_.

The above analysis can be applied to the work of Janssen et al. [5] who reported pooled values for *CRF*_BS_ of 0.90 (0.40–1.41)% for cardiovascular mortality, and 0.68 (0.31–1.06)% for all-cause mortality, per 10 μg m^−3^. The studies from which the pooled estimates are derived relate to BS data from 1986–1996. The analysis in Section 2 suggests a BS:PM_10_ ratio at that time of approximately 0.3 (range 0.2–0.4). So if the toxicity of PM_10_ were contained only within the BS component, *CRF*_PM10_ values of ~0.3% and ~0.2% for cardiovascular and all-cause mortality respectively (per 10 μg m^−3^ PM_10_) would be anticipated. The pooled *CRF*_PM10_ values reported by Janssen et al. [5] are 0.60 (0.23–0.97)% and 0.48 (0.18–0.79)% per 10 μg m^−3^, which in both cases are greater than the *CRF*_PM10_ estimated from consideration of the BS:PM_10_ ratio but less than the *CRF*_BS_. This therefore suggests that there may be a mortality effect associated with other components of PM_10_ to which the BS measurement is insensitive-i.e. non-black components and/or particles larger than the 4.4 μm cut-off of a BS sampler—but that this mortality effect is smaller per μg m^−3^ increment than that associated with the same increment in BS concentration. A similar analysis is not possible between BS and PM_2.5_ since Janssen et al. [5] do not report values of *CRF*_PM2.5_.

As a second example, the cohort study of Yap et al. [10] determined a statistically-significant 5% relative risk in all-cause mortality per 10 μg m^−3^ increment in 1970s decadal-mean BS. If equal toxicity for all particles is assumed, then the *CRF*_PM10_ and *CRF*_PM2.5_ values for long-term all-cause mortality are both also 5% per 10 μg m^−3^ increment in PM_10_ or PM_2.5_. The data presented in Section 2 indicate BS:PM_10_ and BS:PM_2.5_ ratios in the 1970s of ≈ 0.7 and ≈ 1.0 respectively, so if toxicity is assumed associated only with the component to which the BS is sensitive then the *CRF*_BS_ value would equate to *CRF*_PM10_ and *CRF*_PM2.5_ values of 3.5% and 5%, respectively, per 10 μg m^−3^ increment in the PM_10_ and PM_2.5_ prevailing in the 1970s. If the associations of mortality with BS derived for exposures in the 1970s remain valid for exposures to airborne PM around the year 2000 (when BS:PM_10_ and BS:PM_2.5_ ratios were ≈ 0.3 and ≈ 0.5, respectively) then, if toxicity is again assumed only in the BS-sensitive component, the *CRF*_PM10_ and *CRF*_PM2.5_ values would be ≈ 1.5% and ≈ 2.5%, respectively, per 10 μg m^−3^ increment in the PM_10_ and PM_2.5_ prevailing at that time. COMEAP [39] report a *CRF*_PM2.5_ for long-term all-cause mortality of 6%. Comparing this to the effective *CRF*_PM2.5_ of 2.5–3.5% of the Yap et al. [10] study, suggests that the assumption that all the toxicity quantified in the Yap et al. study is associated with what is measured by BS is not valid and that other non-BS components of the inhaled particle mixture also contribute to the associated all-cause mortality. This interpretation is consistent with that described above for the BS-based time-series epidemiology, except that for these long-term studies the mortality effect associated with the non-BS component of PM_2.5_ appears to be at least as large as that for the same increment in BS concentration. If the assumption regarding time-invariant mortality association with BS between 1970s and 2000 is not correct then this particular comparative analysis of CRFs doesn’t hold either.

A BS measurement may be a surrogate for other, or additional, toxic components of the particle mixture, such as transitions metals associated with traffic particle emissions. This would not affect the above analyses of CRF values if BS were a consistent surrogate of these other components.

Ratios of BS to PM_x_ were likely to have varied with location at given time points, as has been noted for relationships between BC, EC and BS [24]. The historic data analysed here were urban background and, for the most part, for the UK. Nevertheless it is emphasised again that, because of this variability, only broad trends in approximate BS:PM_x_ ratios can be derived here. However, epidemiological studies are generally based on measurements at one or a few fixed sites and therefore also do not take into account intra-urban variation. So the analyses presented here of approximate BS:PM_x_ ratios at epidemiologically-relevant monitoring sites is consistent with epidemiology methods; and any ‘sub-population’ intra-urban variability does not negate the potential of deducing information from the relationship between CRFs for BS and for PM_x_ at a given site. Uncertainty is also intrinsically present when an epidemiological study yields a CRF with respect to BS or PM_x_ because it is not possible to know exactly what particle mixture led to the particular BS or PM_x_ values used in the epidemiology.

A further area of uncertainty in interpreting BS data is the extent of any change over time in the specific optical absorption coefficient of the material causing the darkness. In the BS method the reflectance depends on both the size of the black particles (absorption per unit mass increases with decreasing particle) and the dilution of the black particles with non-black particles (absorption is greater for a fixed mass of black particles that is internally or externally mixed with transparent particles) [40–42]. It is probable that both the size distribution and the degree of mixing of black particles changed during the period under consideration. For example, the dominant source of black particles in the latter part of this historic times series is from high-pressure combustion (vehicle engines) which produces particles with smaller diameters than black particles from atmospheric pressure combustion processes (coal and other solid-fuel burning). If in more recent times the black particles are in a smaller size fraction and subject to more dilution with non-absorbing particles, then on both counts reflectance measurements are more sensitive than previously to the black particles present. This implies that more recent BS:PM_x_ ratios are slightly greater than they would be if the nature of the contributing dark particles was the same as in the 1960s and early 1970s. A number of studies have shown that filter darkness at the latter end of the time period under consideration is largely contained within the smaller particle fraction [43–45]. The change in size of black particles does not make any difference to the trends in ratios presented in Fig. 2 or the analyses presented in this discussion; however the reduction with time in the proportion of black particles in the PM mixture may in reality be slightly greater than the reduction in the numerical ratios between BS and PM_x_.

## Conclusions

Examination of the published literature and data has enabled estimates of the changing ratios of BS:PM_10_ and BS:PM_2.5_ to be derived for the period 1970 to the early 2000s when routine measurements of BS were discontinued. These ratios help interpret how health concentration-response functions derived from air pollution epidemiology studies where BS was the measure of exposure relate to concentration-response functions expressed as PM_10_ or PM_2.5_, and, consequently, whether health effects are associated solely with the fine black particles to which the BS measurement is sensitive. Application of this analysis to example published data for short and long-term health effects of particulate matter suggest those studies show there are health effects from non-BS components of PM as well.

## Additional file

Additional file 1: Supplementary Information. (PDF 399 kb)

## Abbreviations

BC: Black carbon
BS: Black smoke
COMEAP: Committee on the Medical Effects of Air Pollutants
CRF: Concentration-response function
EC: Elemental carbon
OECD: Organisation for Economic Co-operation and Development
PM: Particulate matter
PM_10_: Mass concentration of particulate matter with aerodynamic diameter <10 μm
PM_2.5_: Mass concentration of particulate matter with aerodynamic diameter <2.5 μm
TEOM: Tapered element oscillating microbalance
TSP: Total suspended particulate (matter)
UK: United Kingdom
