# Supplementary material for: A chronology of ratios between black smoke and PM10 and PM2.5 in the context of comparison of air pollution epidemiology concentration-response functions
Source: Environ Health. 2017 May 3;16:44. doi: 10.1186/s12940-017-0252-2 (PMC5415716; doi:10.1186/s12940-017-0252-2)

SUPPLEMENTARY INFORMATION

A chronology of ratios between Black Smoke and PM_{10} and $PM_{2.5}$ in the context of comparison of air pollution epidemiology concentration-response functions

Mathew R. Heal[1*] and Iain J. Beverland[2]

[1] *School of Chemistry, University of Edinburgh, David Brewster Road, Edinburgh, EH9 3FJ, UK*

[2] *Department of Civil and Environmental Engineering, University of Strathclyde, James Weir Building, 75 Montrose Street, Glasgow, G1 1XJ, UK*

* Corresponding author

This Supplementary Information provides the detail of the concentrations of particulate matter (PM) chemical composition that underpins the summary data provided in Section 3 of the main paper. As described in the introduction to that section of the paper, it is possible to derive BS:PM_x ratios through estimation of historic PM composition via a simple mass balance model, assuming that most of the PM mass arises from the following components: ammonium sulphate, ammonium nitrate, sodium nitrate, sodium chloride, inorganic 'dust', elemental carbon, organic carbon and water of hydration [1;2]. The reconstruction of the time series of concentrations for each of the major chemical components is described in turn here.

Sulphate. Figure S1 shows the annual average 'aerosol' SO_4^{2-} from all sites in the UK with long-running time series from the mid-1970s to 2001 with data archived at https://uk-air.defra.gov.uk/data/. All sites are rural. Sulphate concentration was 3.8 μg m^{-3} at Eskdalemuir for the period 1973-75 remaining at approximately the same concentration, averaged across all available sites, through to the end of the 1980s (5-year network averages for 1981-85 and 1986-1990 were 3.7 and 3.9 μg m^{-3}). Thereafter, concentrations declined rapidly, with 1991-95 and 1996-2000 5-year network averages of 3.0 and 2.2 μg m^{-3}. Figure S1 shows that background (network-averaged) SO_4^{2-} aerosol declined by only about a factor of 2 over the three decades compared with the greater-than a factor of 10 decline in Black

Smoke (BS) over the same period (Figure 1 of main paper). The decline of only a factor 2 in background SO_4^{2-} also contrasts with the approximate factor of 6 decline in UK SO_2 emissions over the same period (naei.defra.org.uk), and similar magnitude SO_2 emissions reductions elsewhere in Western Europe. This may have resulted from oxidant limitation on the conversion of SO_2 to SO_4^{2-} during the earlier part of the time period, and to increasing concentrations of NH_3 in the latter part of the time period, collectively resulting in greater gas-to-particle conversion as the time period progressed [3].

Additional particle-phase inorganic data are archived only for 5 rural sites in the UK in the early 1980s (https://uk-air.defra.gov.uk/data/). Concentrations were apportioned between fine and coarse fractions, the sum of which can be taken approximately to represent concentrations of the components in PM_{10}. The average concentrations for SO_4^{2-}, NO_3^-, NH_4^+ and Cl^- measured over the period 1981 to 1985 are given in Table S1. The 4.1 μg m^{-3} inter-site average for PM_{10} SO_4^{2-} for 1981-85 for the 5 sites given in Table S1 is broadly consistent with the average bulk SO_4^{2-} concentration of 3.7 μg m^{-3} for the 3 sites with data for the same period shown in Figure S1 (Eskdalemuir, Goonhilly and Stoke Ferry).

Lee et al. [4] report average SO_4^{2-} concentrations of ≈8 μg m^{-3} in the mid-1970s for a site at Chilton in Oxfordshire, declining to ≈5 μg m^{-3} by the start of the 1990s. Other papers reporting historic PM component data for the UK are only for relatively short periods of measurement, as follows. From measurements at a few sites in and around Lancaster, north-west England, for different periods between 1978 and 1982, Harrison and co-workers report mean SO_4^{2-} concentrations in the range 5-9 μg m^{-3} [5-7]. Mean SO_4^{2-} concentration for a few months in 1982 in Leeds was 6.8 μg m^{-3}, of which 85% was in the fine fraction [8]. For the period 1986-1989, for sites in and around Colchester, south-east England, Harrison and co-workers report mean SO_4^{2-} concentrations in the range 3-9 μg m^{-3}, varying with season and location [9;10]. These additional SO_4^{2-} concentrations for the 1970s-1980s tend to be somewhat higher than the rural network averages cited in the preceding paragraph but the latter include urban-centre sites and sites in southern England where background PM concentrations are higher because of greater emissions and closer proximity to continental Europe [11].

In summary, therefore, estimates for historic PM_{10} SO_4^{2-} composition are: 1970s, 4-9 μg m^{-3}; 1980s, 4-9 μg m^{-3}, and late 1990s, 2-4 μg m^{-3}. Since SO_4^{2-} is present almost exclusively as $(NH_4)_2SO_4$ [11;12] a factor 1.38 mass scaling was applied, with a further factor 1.29 to allow for water of hydration [1]. The proportion of the PM_{10} SO_4^{2-} that is within the $PM_{2.5}$ size range is taken to be 80-90% [8;12]. The resulting estimated ranges of mass of $(NH_4)_2SO_4$ in PM_{10} and $PM_{2.5}$ historically are what are summarised in Table 1 of the main paper.

Nitrate. The data from the 5 rural sites in operation as a national network in the early 1980s (Table S1) indicate an average PM_{10} NO_3^- concentration of 1.2 μg m^{-3} and an average split between $PM_{2.5}$ and PM_{10} of 50%. Lee et al. [4] report average bulk particle NO_3^- for the mid-1970s at Chilton of ≈4 μg m^{-3} increasing to ≈5 μg m^{-3} by the start of the 1990s. Mean NO_3^- at sites around Lancaster for periods between 1979 and 1982 ranged between 2.7 and 6.0 μg m^{-3} [5-7]; for Leeds in 1982 it was reported as 2.8 μg m^{-3}, of which 68% was in the fine fraction [8]; and for sites around Essex in the period 1986-89 mean values were in the range 2.7-9.8 μg m^{-3} [9;10]. For the same reasons given above for SO_4^{2-}, these short-campaign NO_3^- concentrations are higher than the rural network values given in Table S1.

In summary, estimates for historic PM_{10} NO_3^- concentration are: 1970s, 2-4 μg m^{-3}; 1980s, 2-6 μg m^{-3}; late 1990s, 2-6 μg m^{-3}. A factor 1.33 was applied to scale NO_3^- to mass of NH_4NO_3 and $NaNO_3$ (the assumed split between the two compounds is not important since the scaling factor is almost the same for each), plus a further factor 1.29 to allow for water of hydration [1]. The proportion of the PM_{10} NO_3^- within $PM_{2.5}$ was taken to be in the range 50-70%. The resulting masses are presented in Table 1 of main paper.

Chloride. Average PM_{10} Cl^- from the rural network measurements in the early 1980s was 1.3 μg m^{-3} (Table S1), with 28% on average in $PM_{2.5}$. For measurements in Leeds in 1982, Cl^- was 1.5 μg m^{-3} with 27% in the fine fraction. For measurements near Lancaster, in the north-west of England and subject to greater marine influence, mean Cl^- was 2.9 μg m^{-3}, with 38% on average in the fine fraction [6]. Likewise, mean Cl^- measured in PM_{10} in the west of Scotland, in 2000, was also higher at ≈2 μg m^{-3} [2].

Since Cl^- in UK PM is largely derived from wind-generated sea-salt particle formation its contribution to PM is not expected to have changed over the time period considered here, aside from a very small decrease resulting from decreased emissions of anthropogenic HCl. The concentration of PM_{10} Cl^- is therefore assumed to be the same for the 1970s, 1980s and late 1990s and taken to be in the range 1.5-2.5 μg m^{-3}. A factor 1.65 is used to scale to the sodium chloride concentrations given in Table 1 of the main paper. The estimate for proportion of PM_{10} Cl^- within $PM_{2.5}$ is 20-40%.

Dust. A major primary component of PM not measured in the datasets cited above is (re)suspended dust. As for marine-derived sea-salt, it is assumed that the contribution of natural dust will not have changed significantly over the years, although there may have been more non-black anthropogenic 'dust' from dustier industrial processes in the 1970s than in the 1990s that may have influenced sites in the vicinity of such processes or in urban areas more generally. It is assumed here that the abundance of this component has historically been approximately the same (≈2-4 μg m^{-3} for PM_{10}) as derived from mass closure methods on direct measurements of Ca and Fe in Birmingham [1] and similarly for measurements in west Scotland in 2000 [2]. For the purpose of allocation to particle size fractions, the proportion of PM_{10} dust within $PM_{2.5}$ is taken to be in the range 20-40% [11].

Elemental and organic carbon. Black smoke values can be used to estimate a mass concentration for the black carbon (BC) constituent of PM through the following expression [13].

$$[BC / \mu g\ m^{-3}] = (0.27 \pm 0.03).BSI_{BRITISH} - (4.0 \pm 0.1)\times 10^{-4}(BSI_{BRITISH})^2$$

For the estimations here it is assumed that elemental carbon (EC) is the same as BC. The data shown in Figure 1 of the main paper indicate 1970s, 1980s and late 1990s UK-network average BS values of ≈35, ≈18 and ≈8 μg m^{-3}, respectively, from which estimates of EC concentrations of 8-10, 4-5 and ≈2-3 μg m^{-3} are derived.

In a review of data from the 1980s and early 1990s QUARG [12] reported that organic carbon (OC) normally constituted 60-80% of total carbon (OC + EC). Applying a wider range of 50-80% for the fraction OC/(OC + EC) to the EC estimates, to allow for EC comprising a greater proportion of carbonaceous material, yields estimates for OC concentrations in the 1970s, 1980s and late 1990s of 9-25, 4-12 and 2-6 μg m^{-3}, respectively. The OC to organic material

(OM) scaling factor varies with the nature of the OM, ranging from about 1.2 for primary OM close to emission source to about 1.8-2.0 for biomass/biogenic OM [14;15]. The appropriate OC to OM scaling factor is the weighted average for the contributing sources to the given PM size fraction, but it will have a value between the above extremes. Historically, primary sources were likely the more dominant contribution to OM. Therefore an OC to OM scaling factor range 1.3-1.7 was used to derive the historic OM concentration ranges shown in Table 1 of the main paper. To derive ranges for quantities calculated by combining other quantities with ranges, standard formulae for combining uncertainties were used with the assumption that an individual range corresponded to ±2 sd of uncertainty (i.e. approximately equivalent to a 95% confidence interval). Since carbonaceous material is predominantly present in the fine fraction, the assumed ranges for calculation of proportion of EC and OC in $PM_{2.5}$ were both 70-90%.

Total speciated PM_x concentrations.

The estimates for the sums of the historical component masses in $PM_{2.5}$ and PM_{10} are also presented in Table 1 of the main paper.

References

1. Harrison RM, Jones AM, Lawrence RG. A pragmatic mass closure model for airborne particulate matter at urban background and roadside sites. Atmos Environ. 2003;37:4927-33.

2. Gibson MD, Heal MR, Bache D, Hursthouse AS, Beverland IJ, Craig SE, Clark CF, Jackson MH, Guernsey JR, Jones C. Using mass reconstruction along a four-site transect as a method to interpret PM10 in West-Central Scotland, United Kingdom. J Air Waste Manage Assoc. 2009;59:1429-36.

3. Harrison RM, Stedman J, Derwent D. New directions: Why are PM10 concentrations in Europe not falling? Atmos Environ. 2008;42:603-6.

4. Lee DS, Dollard GJ, Derwent RG, Pepler S. Observations on gaseous and aerosols components of the atmosphere and their relationships. Water Air Soil Poll. 1999;113:175-202.

5. Harrison RM, Pio CA. Major ion composition and chemical associations of inorganic atmospheric aerosols. Environ Sci Technol. 1983;17:169-74.

6. Harrison RM, Pio CA. Size-differentiated composition of inorganic atmospheric aerosols of both marine and polluted continental origin. Atmos Environ. 1983;17:1733-8.

7. Colbeck I, Harrison RM. Ozone secondary aerosol visibility relationships in northwest England. Sci Total Environ. 1984;34:87-100.

8. Clarke AG, Willison MJ, Zeki EM. A comparison of urban and rural aerosol composition using dichotomous samplers. Atmos Environ. 1984;18:1767-75.

9. Harrison RM, Allen AG. Measurements of atmospheric HNO3, HCl and associated species on a small network in eastern England. Atmospheric Environment Part A-General Topics. 1990;24:369-76.

10. Kitto AMN, Harrison RM. Processes affecting concentrations of aerosol strong acidity at sites in eastern England. Atmospheric Environment Part A-General Topics. 1992;26:2389-99.

11. AQEG. Particulate Matter in the United Kingdom. Second report of the Air Quality Expert Group. UK Department for Environment, Food and Rural Affairs, London. PB10580. http://uk-air.defra.gov.uk/library/reports?report_id=269; 2005.

12. QUARG. Airborne particulate matter in the United Kingdom. Third Report of the Quality of Urban Air Review Group. UK Department of Environment, London. ISBN 0 952077132. http://uk-air.defra.gov.uk/library/reports?report_id=48; 1996.

13. Heal MR, Quincey P. The relationship between black carbon concentration and black smoke: a more general approach. Atmos Environ. 2012;54:538-44.

14. Heal MR, Naysmith P, Cook GT, Xu S, Raventos Duran T, Harrison RM. Application of 14C analyses to source apportionment of carbonaceous PM2.5 in the UK . Atmospheric Environment. 2011;45:2341-8.

15. Pio C, Cerqueira M, Harrison RM, Nunes T, Mirante F, Alves C, Oliveira C, Sanchez de la Campa A, Artíñano B, Matos M. OC/EC ratio observations in Europe: Re-thinking the approach for apportionment between primary and secondary organic carbon. Atmos Environ. 2011;45:6121-32.

Supplementary Information Table

Table S1: Summary of average concentrations of inorganic components within $PM_{2.5}$ and PM_{10} from daily measurements at 5 rural locations in the UK between 1981 and 1985. Data from https://uk-air.defra.gov.uk/data/. Note there were considerable missing daily data within this period which may have biased average values by an unquantifiable amount.

		**Eskdalemuir**	**Goonhilly**	**Lough Navar**	**Ludlow**	**Stoke Ferry**	**Average across sites**
Within $PM_{2.5}$ / µg m^{-3}	SO_4^{2-}	3.13	3.77	1.86	4.38	4.67	3.56
	NO_3^-	0.43	0.55	0.41	0.78	0.97	0.63
	NH_4^+	0.99	1.39	0.49	1.95	1.58	1.28
	Cl–	0.36	0.58	0.36	0.24	0.21	0.35
Within PM_{10} / µg m^{-3}	SO_4^{2-}	3.59	4.33	2.22	4.88	5.24	4.05
	NO_3^-	0.94	1.28	0.89	1.40	1.59	1.22
	NH_4^+	1.15	1.52	0.60	2.09	1.75	1.42
	Cl–	1.08	2.20	1.23	0.92	0.82	1.25
$PM_{2.5}$:PM_{10} / %	SO_4^{2-}	87.2	87.0	83.8	89.8	89.1	87.4
	NO_3^-	46.0	43.0	46.4	55.9	60.7	50.4
	NH_4^+	86.8	91.3	82.0	93.2	90.2	88.7
	Cl–	32.9	26.3	29.2	26.3	25.1	28.0

Supplementary Information Figure

Figure S1: Time series of annual average particle sulphate measured at 9 rural locations in the UK. Data from https://uk-air.defra.gov.uk/data/. Annual averages were calculated only when >50% of daily values were available. Missing data may bias average values by an unquantifiable amount. The solid black line shows the rolling 3-year mean of the plotted data.

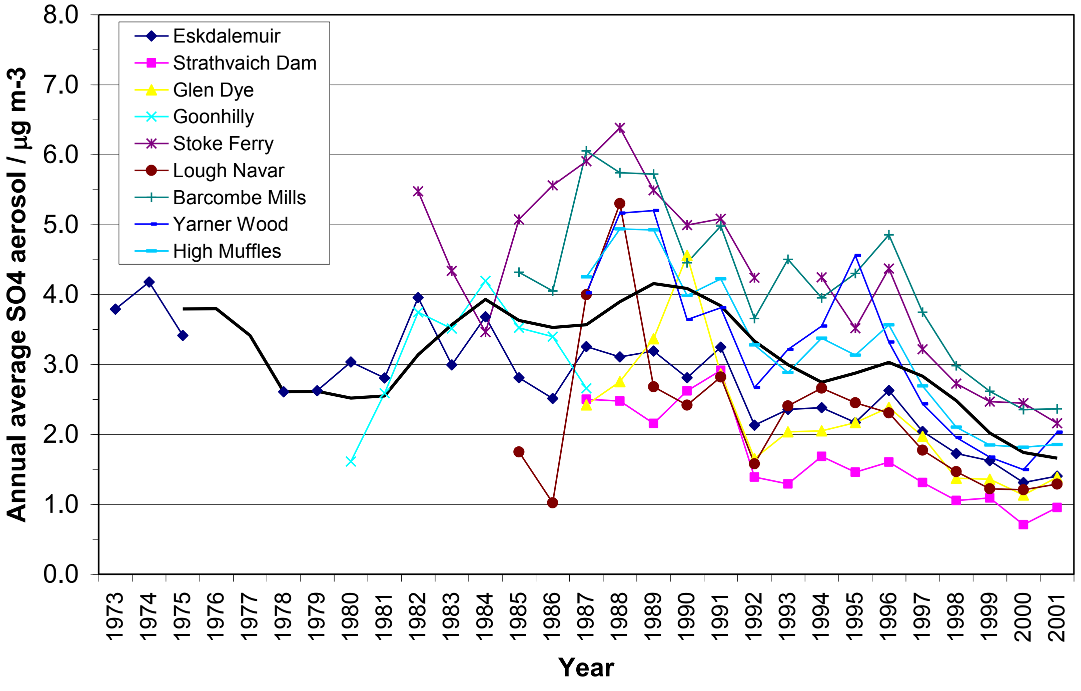

Supplement: Additional file 1: — Supplementary Information. (PDF 399 kb) [file 12940_2017_252_MOESM1_ESM.pdf]
